# Medical Expectations of Physicians on AI Solutions in Daily Practice: Cross-Sectional Survey Study

**DOI:** 10.2196/50803

**Published:** 2024-03-25

**Authors:** Mara Giavina-Bianchi, Edson Amaro Jr, Birajara Soares Machado

**Affiliations:** 1 Big Data Department, Hospital Israelita Albert Einstein, Sao Paulo, Brazil

**Keywords:** artificial intelligence, adoption, AI, acceptance, opinion, perceptions, survey, expectations, physician, medical survey, qualitative study

## Abstract

**Background:**

The use of artificial intelligence (AI) in medicine has been a trending subject in the past few years. Although not frequently used in daily practice yet, it brings along many expectations, doubts, and fears for physicians. Surveys can be used to help understand this situation.

**Objective:**

This study aimed to explore the degree of knowledge, expectations, and fears on possible AI use by physicians in daily practice, according to sex and time since graduation.

**Methods:**

An electronic survey was sent to physicians of a large hospital in Brazil, from August to September 2022.

**Results:**

A total of 164 physicians responded to our survey. Overall, 54.3% (89/164) of physicians considered themselves to have an intermediate knowledge of AI, and 78.5% (128/163) believed that AI should be regulated by a governmental agency. If AI solutions were reliable, fast, and available, 77.9% (127/163) intended to frequently or always use AI for diagnosis (143/164, 87.2%), management (140/164, 85.4%), or exams interpretation (150/164, 91.5%), but their approvals for AI when used by other health professionals (85/163, 52.1%) or directly by patients (82/162, 50.6%) were not as high. The main benefit would be increasing the speed for diagnosis and management (106/163, 61.3%), and the worst issue would be to over rely on AI and lose medical skills (118/163, 72.4%). Physicians believed that AI would be useful (106/163, 65%), facilitate their work (140/153, 91.5%), not alter the number of appointments (80/162, 49.4%), not interfere in their financial gain (94/162, 58%), and not replace their jobs but be an additional source of information (104/162, 64.2%). In case of disagreement between AI and physicians, most (108/159, 67.9%) answered that a third opinion should be requested. Physicians with ≤10 years since graduation would adopt AI solutions more frequently than those with >20 years since graduation (*P*=.04), and female physicians were more receptive to other hospital staff using AI than male physicians (*P*=.008).

**Conclusions:**

Physicians were shown to have good expectations regarding the use of AI in medicine when they apply it themselves, but not when used by others. They also intend to use it, as long as it was approved by a regulatory agency. Although there was hope for a beneficial impact of AI on health care, it also brings specific concerns.

## Introduction

The use of artificial intelligence (AI) is expanding throughout the field of medicine, driven by researchers and entrepreneurs [1,2]. Over the last decade, the number of publications on AI in medicine and biomedicine has substantially increased [3]. AI solutions might change the clinical practice in nearly all medical disciplines and areas of health care. Despite the potential of machine learning to improve multiple aspects of patient care, there are still barriers to clinical adoption. Important questions remain regarding how machine learning interventions are being incorporated into health care [4]. A reluctance to adopt AI-based solutions might be due to a lack of knowledge; fear of error; and concerns about losing jobs, power, or both [5]. Another perceived limitation of AI applications is the belief that communication and empathy are human competencies that cannot be replaced by AI. In addition, the ability to provide value-based care requires the physicians’ judgments. Some possible benefits included expectations about improved efficiencies, especially with respect to the reduction of administrative burdens on physicians [6]. Examples of practical use of AI solutions in clinical routine are still scarce around the globe [2]. The increasing development in medical systems using AI brings enormous expectations and fears for both physicians and patients.

Physicians are likely to be the “earliest” adopters of AI solutions for patient care and inevitably should become direct AI operators. Therefore, they play a pivotal role in the acceptance and implementation of clinical AI, and consequently, their views need to be known, explored, and understood [7]. Opinion surveys are important tools in assessing satisfaction with a particular service and consist of a list of questions whose objective is to extract certain data from a group of people [8]. Previous studies on the acceptance of the use of AI in medicine were limited to specific areas, such as radiology [5,9-11], dermatology [1,12-14], and ophthalmology [15-17], as well as to specific countries. However, at the time of writing, we were unable to find studies exploring this subject among Brazilian physicians, and there are very few studies in Latin America, leaving a gap in this part of the globe. Is the perception of AI adoption similar to other countries? At the same time, some aspects are not yet explored—does the acceptance of AI solutions vary according to the number of years since medical graduation or the physician’s sex? Routine observation could indicate that younger individuals are keener to accept new technologies, and no information regarding sex preferences on this matter has yet been evaluated, as far as we know.

The main objective of this study was to assess the expectations, fears, and thoughts of physicians from a single-center private hospital about some practical aspects of the hypothetical use of AI solutions in medical daily practice. The secondary objective was to verify if there were differences in the opinions among physicians according to the sex and time since graduation.

## Methods

### Ethical Considerations

This study was approved by the Ethics Committee of Hospital Israelita Albert Einstein (CAAE: 30749620.6.0000.0071). All participants provided informed consent, and the collected data were anonymized. There were no financial incentives for answering the questionnaire.

### Study Design and Target Population

We performed a cross-section observational study via an opinion survey. Our target population was physicians who were part of the clinical staff of the Hospital Israelita Albert Einstein (HIAE). The survey was sent to 7457 physicians from the clinical staff with an email address registered in the HIAE marketing department. There were no exclusion criteria, and responses were collected from August 1 to October 1, 2022 (60 days). The HIAE is a nonprofit, public interest organization based in São Paulo, Brazil. Although physicians of the HIAE do not represent the entire population of physicians in Brazil, their answers can give some insights on the subject, since, at this moment, we have none.

### Questionnaire

The questionnaire was composed of 30 questions. Question 1 was the informed consent form (ICF). In case of acceptance of the ICF, the next questions were presented to the individual. If not, the survey would be terminated. The time to complete the questionnaire was approximately 7 minutes on average. The survey was completely anonymous and confidential, and only the authors of this work had access to the answers. The complete questionnaire can be viewed in Multimedia Appendix 1. The questions were developed by the authors based on their experience in developing AI solutions for physicians and on previous published medical literature.

No specific technology was evaluated. Most of the questions asked about the use of AI algorithms for the diagnosis or management of diseases, aiming to capture the possible expectations of our target population of physicians in clinical practice. The questionnaire was divided into 5 sections. Section 1 was the ICF (question 1). Section 2 (questions 2-12) was designed to profile the physicians, including sex; age; highest level of education; medical specialty; years since graduation; private versus public sector work; city and state of work; self-assessment knowledge of AI in general (not specific for health care AI solutions); and the use of computer or smartphone applications that use AI solutions for daily tasks, such as WhatsApp, Instagram, Facebook, Waze, Google Maps, and bank apps, among others. We did not ask specifically about the use of AI solutions for health care in their daily work, only if they were aware of AI solutions in medicine. Section 3 (questions 13-18) explored the physicians’ thoughts about AI solutions for diagnosis, management, subsidiary exams, and interpretation of diseases, such as COVID-19, and about the use of AI solutions for the diagnosis or treatment of diseases by nurses or physiotherapists or directly by the patient. We also proposed a hypothetical exercise to evaluate physicians’ level of anxiety and actions taken if they had received a suspicious diagnosis of melanoma for 1 of their skin lesions by an AI algorithm. Section 4 (questions 19-24) asked about expected benefits and problems, possible frequency of AI adoption, workload, and utility. Section 5 (questions 25-30) were about physicians’ replacement by AI solutions, financial expectations, possible scenarios of disagreements between AI and physicians, and legal and regulatory aspects. Along with the questions, there were many opportunities for physicians to provide comments about the answers in an open-text box. Physicians could skip any question; thus, the number of responders could vary among the questions.

### Emailing the Questionnaire

The survey was sent by email to all physicians with an email address linked to the HIAE. In the first email, sent on August 1, 2022, a brief introduction inviting the physician to participate in the survey and the questionnaire link to be completed in the SurveyMonkey computer program (SurveyMonkey Inc) [18] were sent to all physicians. In the second email, sent on August 8, 2022, we replicated the same email to those who had not completed the survey from the previous email. There was a third email sent on September 5, 2022, to the remaining individuals. The survey ended on September 30, 2022. This time frame of 60 days and the number of reminders during the period followed the current modus operandi of the hospital’s marketing department for all studies sent electronically. SurveyMonkey’s program has a blocking mechanism that prevents the same participant from responding to the survey more than once. It identifies and notifies the user that the questionnaire had already been answered, blocking a new response. The survey was previously tested by 3 physicians of the HIAE medical team who were part of the target population. Our work followed the CROSS (Checklist for Reporting Survey Studies) [4].

### Statistical Analysis

The response rate was the number of physicians who responded the questionnaire divided by the number of physicians to whom the email was sent multiplied by 100. The completion rate was the number of surveys answered and sent divided by the number of surveys initiated by respondents multiplied by 100. There were 2 different questions involving statistical analysis. In the first question—“does the time since medical graduation matter?”—the participants were divided into 3 groups (≤10 years, 11-20 years, or >20 years) according to their answer to question 7 of the questionnaire. In the second question—“does the subject’s sex matter?”—physicians were divided into male or female individuals according to their answer to question 3. Statistical analyses between for both analyses were performed using the *χ*^2^ test in Prism software (version 6; GraphPad Software Inc). *P* values <.05 were considered significant.

## Results

### Participant Characteristics

In all, 181 physicians accepted the ICF. The response rate was 2.4% (181/7457). The completion rate of the questionnaire was 90.6% (164/181). As physicians could skip any question, the number of responders could vary among the questions.

Table 1 shows the profile of the physicians who responded to the survey. They were mostly male (111/171, 64.9%), 36-55 years of age (99/171, 57.9%), and with >20 years since medical graduation (110/171, 64.3%). As for the place of work, 68.4% (117/171) worked mainly in the private sector, 26.9% (46/171) worked equally in both sectors, and 5.3% (9/171) worked mainly in the public sector. All academic degrees were present in the study; most had a residency or specialization internship (57/171, 33.3%), doctorate degree (55/171, 32.2%), or master’s degree (43/171, 25.1%). The distribution among specialties was heterogeneous and skewed: surgeons were the most frequent (37/169, 21.9%). This finding may reflect the different number of physicians of each specialty linked to the hospital mailing list. There were probably many more pediatricians in the hospital’s mailing list than psychiatrists. Almost all (166/171, 97%) of them worked in São Paulo city, as the main hospital is in this location. Most (89/164, 54.3%) of them classified their own knowledge of AI as intermediate. Most of the participants used smartphones or computers applications that incorporate AI algorithms for daily tasks outside of work (119/164, 72.6%) and claimed to be aware of AI algorithms applied specifically to medicine (86/164, 52.4%). There were no statistical differences among the participants based on sex. For the group**s based on** “years since **graduation,**” there was a significant *P* value (*P*=.049) in the self-assessment of AI knowledge.

**Table 1.** Profile of physicians who answered the opinion questionnaire on artificial intelligence (AI) solutions at the Hospital Israelita Albert Einstein (questions 2-12).

Questions and responses		Years since graduation, n (%)			Sex, n (%)		Total (n=171), n (%)
		≤10 (n=13)	11-20 (n=48)	>20 (n=110)	Male (n=111)	Female (n=60)	
**2. Sex**							
	Female	6 (46)	18 (38)	36 (33)	0 (0)	60 (100)	60 (35)
	Male	7 (54)	30 (63)	74 (67)	111 (100)	0 (0)	111 (65)
**3. Age range (years)**							
	26-35	11 (85)	2 (4)	0 (0)	9 (8)	4 (7)	13 (8)
	36-45	2 (15)	43 (90)	3 (3)	29 (26)	19 (32)	48 (28)
	46-55	0 (0)	3 (6)	48 (44)	29 (26)	22 (37)	51 (30)
	56-65	0 (0)	0 (0)	33 (30)	21 (19)	12 (20)	33 (19)
	>65	0 (0)	0 (0)	26 (24)	23 (21)	3 (5)	26 (15)
**4. Highest academic degree (>20 years since graduation: n=111)**							
	Medical	0 (0)	0 (0)	1 (1)	1 (1)	0 (0)	1 (1)
	Residency or specialization internship	10 (77)	25 (52)	22 (20)	35 (32)	22 (37)	57 (33)
	Master’s degree	3 (23)	13 (27)	27 (24)	27 (24)	16 (27)	43 (25)
	Doctorate degree	0 (0)	7 (15)	48 (43)	36 (32)	18 (30)	54 (32)
	Postdoctoral research	0 (0)	3 (6)	6 (5)	6 (5)	3 (5)	9 (5)
	Associated professor	0 (0)	0 (0)	6 (5)	6 (5)	0 (0)	6 (4)
	Other	0 (0)	0 (0)	1 (1)	0 (0)	1 (2)	1 (1)
**5. Specialty (>20 years since graduation: n=108; male: n=57; female: n=168)**							
	Dermatology	0 (0)	0 (0)	3 (3)	0 (0)	3 (5)	3 (2)
	Gynecology or obstetrics	0 (0)	1 (2)	16 (15)	9 (8)	8 (14)	17 (10)
	Internal medicine	2 (2)	11 (23)	12 (11)	14 (13)	11 (19)	25 (15)
	Management	1 (17)	4 (8)	1 (1)	5 (5)	1 (2)	6 (4)
	Ophthalmology	0 (0)	0 (0)	3 (3)	3 (3)	0 (0)	3 (2)
	Orthopedics	0 (0)	3 (6)	7 (6)	10 (9)	0 (0)	10 (6)
	Otorhinolaryngology	0 (0)	0 (0)	5 (5)	4 (4)	1 (2)	5 (3)
	Other	4 (33)	12 (25)	17 (16)	18 (16)	14 (24)	32 (19)
	Pathology	0 (0)	0 (0)	2 (2)	1 (1)	1 (1)	2 (1)
	Pediatrics	4 (33)	6 (0)	12 (11)	10 (9)	12 (20)	22 (13)
	Psychiatry	0 (0)	0 (0)	2 (2)	0 (0)	2 (3)	2 (1)
	Radiology	0 (0)	3 (6)	1 (1)	2 (2)	2 (3)	4 (2)
	Surgery	2 (17)	8 (17)	27 (25)	35 (32)	2 (7)	37 (22)
**6. Years since graduation**							
	5-10	13 (100)	0 (0)	0 (0)	7 (6)	6 (10)	13 (8)
	11-20	0 (0)	48 (100)	0 (0)	30 (27)	18 (30)	48 (28)
	>20	0 (0)	0 (0)	110 (100)	74 (67)	36 (60)	110 (64)
**7. Work sector**							
	Mostly public	1 (1)	3 (63)	5 (5)	4 (4)	5 (8)	9 (5)
	Mostly private	8 (62)	35 (73)	73 (66)	77 (69)	39 (65)	116 (68)
	Equally in both	4 (31)	10 (21)	32 (29)	30 (27)	16 (27)	46 (27)
**8. Workplace (state; male: n=110; female: n=59; total: n=169)**							
	São Paulo	13 (100)	48 (100)	108 (98)	108 (98)	59 (100)	167 (99)
	Other	0 (0)	0 (0)	2 (2)	2 (2)	0 (0)	2 (1)
**9. Workplace (city; >20 years since graduation: n=111)**							
	Capital	13 (100)	48 (100)	105 (95)	107 (96)	58 (97)	165 (96)
	Coast or inland	0 (0)	0 (0)	6 (5)	4 (4)	2 (3)	6 (4)
**10. Self-assessment of the degree of AI knowledge in general** **(years since graduation, <10: n=12; 11-20: n=46; >20: n=106; male: n=108; female: n=55; total: n=163)^a^**							
	Low	0 (0)	19 (41)	30 (28)	27 (25)	21 (38)	48 (29)
	Medium	8 (67)	20 (43)	61 (58)	60 (56)	29 (53)	89 (55)
	High	4 (33)	7 (15)	12 (11)	19 (18)	4 (7)	23 (14)
	None	0 (0)	0 (0)	3 (3)	2 (2)	1 (2)	3 (2)
**11. Frequency of AI use for daily life activities (years since graduation, <10: n=12; 11-20: n=45; >20: n=103; male: n=105; female: n=53; total: n=158)**							
	Never	0 (0)	0 (0)	1 (1)	0 (0)	1 (2)	2 (1)
	Rarely	1 (8)	1 (2)	2 (2)	3 (28)	1 (2)	4 (2)
	Sometimes	1 (8)	11 (24)	22 (21)	22 (20)	12 (22)	34 (21)
	Frequently	5 (42)	21 (46)	49 (46)	48 (44)	26 (47)	74 (45)
	Always	5 (42)	12 (26)	28 (26)	31 (29)	14 (25)	45 (27)
	Do not know	0 (0)	1 (2)	4 (4)	4 (4)	1 (2)	5 (3)
**12. Aware of AI solutions in medicine? (years since graduation, <10: n=12; 11-20: n=46; >20: n=106; male: n=108; female: n=55; total: n=163)**							
	Yes	11 (92)	24 (52)	51 (48)	56 (52)	29 (53)	85 (52)
	No	0 (0)	13 (28)	35 (33)	34 (31)	14 (25)	48 (29)
	Uncertain	1 (8)	9 (20)	20 (19)	18 (17)	12 (22)	30 (18)

a *P*=.049 when comparing groups based on years since graduation.

### The Use of AI Solutions

**Figure 1.**
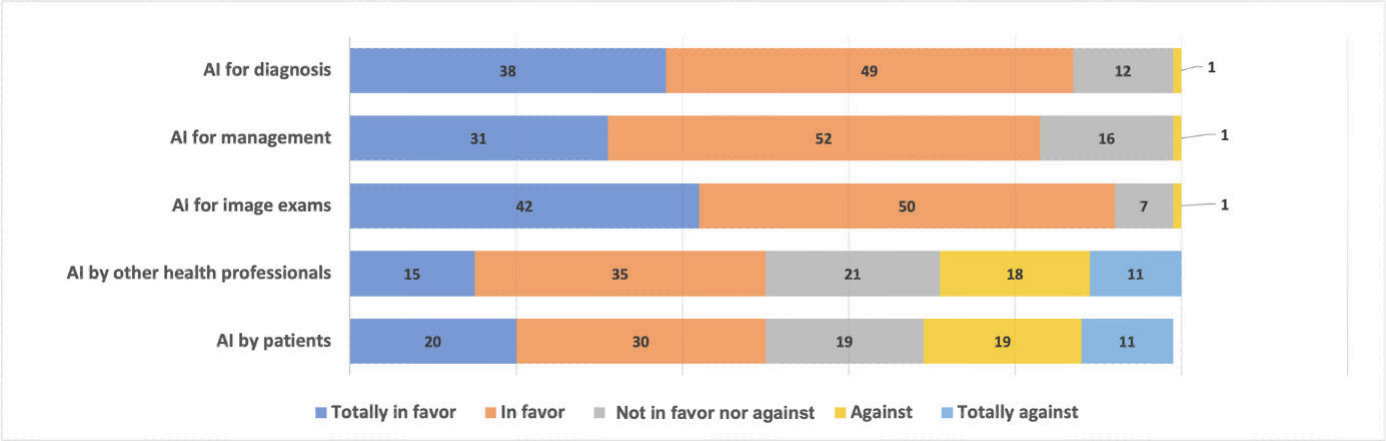
Physicians’ expectations about the role of artificial intelligence (AI) solutions when used by themselves, other health care professionals, or patients (questions 13-17).

In total, 164 participants answered all questions until the end of the questionnaire. Figure 1 summarizes the answers from questions 13-17 of the survey, and Table 2 shows the answers to questions 13-17 according to the studied groups. For physicians in general, there was a belief that AI algorithms would be helpful for patients’ diagnosis (143/164, 87.2%) and management (140/164, 85.5%) and would support image exams interpretation (150/164, 91.5%) when used by them. They were divided about the use of AI by other health professionals, such as nurses or physiotherapists (85/163, 52.1% approved), or by the patient themselves (82/162, 50.6% approved).

**Table 2.** Physicians’ expectations about the role of artificial intelligence (AI) solutions when used by themselves, other health care professionals, or patients (questions 13-17), according to years since graduation and sex.

Questions and responses		Years since graduation n (%)			Sex n (%)	
		≤10 (n=12)	11-20 (n=46)	>20 (n=106)	Male (n=108)	Female (n=55)
**13. Use of AI to support diagnosis by physicians (eg, COVID-19)**						
	Totally in favor	7 (58)	15 (33)	41 (39)	45 (42)	18 (33)
	In favor	4 (33)	23 (50)	53 (50)	50 (46)	29 (53)
	Not in favor nor against	0 (0)	8 (17)	11 (10)	11 (10)	8 (15)
	Against	1 (8)	0 (0)	1 (1)	2 (2)	0 (0)
	Totally against	0 (0)	0 (0)	0 (0)	0 (0)	0 (0)
**14. Use of AI to support disease management by physicians (eg, corticosteroids for COVID-19)**						
	Totally in favor	5 (42)	13 (28)	33 (31)	37 (34)	14 (25)
	In favor	6 (50)	22 (48)	57 (54)	54 (50)	30 (55)
	Not in favor nor against	1 (8)	10 (22)	15 (14)	15 (14)	11 (20)
	Against	0 (0)	1 (2)	1 (1)	2 (2)	0 (0)
	Totally Against	0 (0)	0 (0)	0 (0)	0 (0)	0 (0)
**15. Use of AI to support exam interpretation (eg, x-rays for SARS)**						
	Totally in favor	8 (67)	19 (41)	42 (40)	49 (45)	20 (36)
	In favor	3 (25)	23 (50)	55 (52)	47 (44)	33 (60)
	Not in favor nor against	0 (0)	4 (9)	8 (8)	10 (9)	2 (4)
	Against	1 (8)	0 (0)	1 (1)	2 (2)	0 (0)
	Totally against	0 (0)	0 (0)	0 (0)	0 (0)	0 (0)
**16. Use of AI to aid diagnosis or management by other hospital staff (nurses or physiotherapists** **; >20 years since graduation: n=105; male: n=107)^a^**						
	Totally in favor	3 (25)	8 (17)	14 (13)	18 (17)	7 (13)
	In favor	6 (50)	14 (30)	37 (35)	29 (27)	28 (51)
	Not in favor nor against	2 (17)	10 (22)	22 (21)	21 (20)	12 (22)
	Against	0 (0)	8 (17)	22 (21)	23 (21)	7 (13)
	Totally against	1 (8)	6 (13)	10 (10)	16 (15)	1 (2)
**17. Use of AI directly by patients to aid diagnosis or management (years since graduation, <10: n=10; 11-20: n=47; >20: n=107; male: n=107; female: n=54)**						
	Totally in favor	5 (42)	7 (15)	21 (21)	24 (22)	9 (17)
	In favor	2 (17)	15 (33)	32 (31)	30 (28)	19 (35)
	Not in favor nor against	2 (17)	10 (22)	22 (21)	15 (14)	16 (30)
	Against	0 (0)	8 (17)	22 (21)	23 (21)	7 (13)
	Totally against	1 (8)	7 (15)	10 (10)	15 (14)	3 (6)

a *P*=.008 when comparing male and female physicians.

Interestingly, there was a significant difference in the use of AI solutions by other hospital professionals according to sex: female physicians were more favorable toward it than male physicians (*P*=.008).

When placing themselves in the role of a patient, physicians acknowledged that AI diagnosis solutions when used by nonspecialists might cause distress about some types of diagnosis, such as skin melanoma (question 18). This question asked that if a melanoma detection solution was used by the physicians on themselves regarding a certain lesion and the AI showed a high probability of melanoma diagnosis, what that situation would elicit in their feelings (the degree of anxiety or none) and what action would be taken (how quick they would seek a specialist appointment or if they would not seek one). Overall, 91% (142/156) reported that they would be at least anxious and seek an appointment as soon as possible. The major benefits cited by the physicians were greater speed for diagnosis and management (104/432, 24.1% of responses and 104/164, 63.4% of responders), greater accuracy (84/432, 19.4%), health care cost reduction (84/432, 19.4%), and greater access (67/432, 15.5%). The main issues listed were the fear of relying excessively on the AI algorithms and causing physicians to lose their medical skills (115/447, 25.7% of responses and 115/164, 70.1% of responders), wrongful diagnostic or management reports (84/447, 18.8%), and increasing the distance in the physician-patient relationship (84/447, 18.8%). The intention to adopt AI solutions showed a significant difference among the groups based on years since graduation (*P*=.04). Individuals with ≤10 years since graduation would use AI most of the times or always (10/12, 83%) in greater number than the other 2 groups: 30% (14/46) and 32.4% (34/105) for the groups with 11-20 and >20 years since graduation, respectively (Table 3). When the 3 groups were tested separately, 2 by 2, there was a significant *P* value of .004 when comparing ≤10 and >20 years since graduation and a *P* value of .02 when comparing ≤10 and 11-20 years since graduation, proving that the difference was between the group with ≤10 years since graduation and the other 2 groups. When comparing the groups with 11-20 and >20 years since graduation, the *P* value was .95 for that same question.

**Table 3.** Overview and expectations, frequency of adoption, work facilitation, number of appointments, and utility about artificial intelligence (AI) solutions according to years since graduation and sex (questions 18-24).

Questions and responses		Years since graduation, n (%)			Sex, n (%)	
		≤10 (n=12)	11-20 (n=46)	>20 (n=105)	Male (n=108)	Female (n=54)
**18. Consequences of AI diagnosis if you were a patient (eg, AI solution made a diagnosis of suspicious melanoma for 1 of your skin lesions; years since graduation, 11-20: n=44; >20: n=104; male: n=106; female: n=53)**						
	Extremely anxious and immediate appointment	10 (83)	26 (59)	64 (62)	63 (59)	36 (68)
	Anxious and appointment whenever possible	2 (17)	13 (30)	28 (77)	32 (30)	11 (21)
	Not shaken and appointment whenever possible	0 (0)	4 (9)	9 (9)	11 (10)	2 (38)
	Not shaken and no appointment	0 (0)	1 (2)	0 (0)	0 (0)	1 (2)
	I am a dermatologist	0 (0)	0 (0)	3 (3)	0 (0)	3 (6)
**19. Expected benefits of AI (physicians could pick up to 3 options; years since graduation, <10: n=33; 11-20: n=131; >20: n=285; male: n=291; female: n=141)**						
	Greater speed	9 (27)	33 (25)	64 (22)	67 (23)	37 (26)
	Greater accuracy	6 (18)	27 (21)	54 (19)	58 (20)	26 (18)
	Cost reduction	5 (15)	24 (18)	58 (20)	57 (20)	27 (19)
	Reduction in the number of subsidiary exams	5 (15)	16 (12)	34 (12)	35 (12)	18 (13)
	Reduction in patient anxiety	0 (0)	1 (1)	8 (3)	7 (2)	1 (1)
	Greater access to health care	7 (21)	20 (15)	42 (15)	43 (15)	24 (17)
	Greater patient participation in health care	1 (3)	8 (6)	22 (8)	20 (7)	8 (6)
	Other	0 (0)	2 (2)	3 (1)	4 (14)	0 (0)
**20. Expected problems of AI (physicians could pick up to 3 options; years since graduation, <10: n=33; 11-20: n=134; >20: n=295; male: n=305; female: n=142)**						
	Confidentiality issues	4 (12)	7 (5)	20 (7)	17 (6)	12 (8)
	Worsening of the physician-patient relationship	4 (12)	23 (17)	59 (20)	57 (19)	27 (19)
	Wrongful use of patient’s information by employers and insurance companies	7 (21)	14 (10)	47 (16)	47 (15)	19 (13)
	Errors in diagnosis or management	5 (15)	26 (19)	55 (19)	55 (18)	29 (20)
	Physicians relying too much on AI and losing medical skills	9 (27)	35 (26)	74 (25)	78 (26)	37 (26)
	Increase in health care cost	1 (3)	8 (6)	10 (3)	13 (4)	4 (3)
	Lack of AI transparency	2 (6)	15 (11)	28 (9)	31 (10)	12 (8)
	Other	1 (3)	6 (4)	2 (1)	7 (2)	2 (1)
**21. Expected frequency of AI adoption if the algorithm was reliable and only needed up to 2 min to provide an answer** ^**a**^						
	Never	0 (0)	0 (0)	1 (1)	1 (1)	0 (0)
	Rarely	1 (8)	1 (2)	1 (1)	3 (3)	0 (0)
	Sometimes	1 (8)	10 (22)	20 (19)	18 (17)	13 (24)
	Frequently	0 (0)	21 (46)	48 (46)	44 (41)	24 (44)
	Most of the times	4 (33)	6 (13)	17 (16)	19 (18)	8 (15)
	Always	6 (50)	8 (17)	17 (16)	22 (41)	9 (17)
	Do not know	0 (0)	0 (0)	1 (1)	1 (1)	0 (0)
**22. Work facilitation by AI (assuming the conditions in question 21; years since graduation, <10: n=11; 11-20: n=73; >20: n=104; male: n=106)**						
	Makes work easier	9 (82)	42 (91)	89 (86)	92 (87)	47 (87)
	Makes work more difficult	0 (0)	0 (0)	0 (0)	0 (0)	0 (0)
	Does not change	2 (18)	2 (4)	9 (9)	9 (8)	4 (7)
	Do not know	0 (0)	29 (4)	6 (6)	5 (5)	3 (6)
**23. Number of daily appointments (assuming the conditions in question 21)**						
	Increases	6 (50)	14 (30)	34 (32)	33 (31)	20 (37)
	Decreases	0 (0)	2 (4)	7 (7)	5 (5)	4 (7)
	Stays the same	5 (42)	24 (52)	51 (49)	55 (51)	25 (46)
	Do not know	1 (8)	6 (13)	13 (12)	15 (14)	5 (9)
**24. AI utility in daily work (assuming the conditions in question 21)**						
	Useful for diagnosis	2 (17)	15 (33)	21 (20)	25 (23)	13 (24)
	Useful for management	0 (0)	1 (2)	9 (9)	8 (7)	2 (4)
	Useful for diagnosis and management	9 (75)	27 (59)	70 (67)	68 (63)	38 (70)
	Not help nor hinder	0 (0)	0 (0)	2 (2)	2 (2)	0 (0)
	Hinders	1 (8)	1 (2)	0 (0)	2 (2)	0 (0)
	Do not know	0 (0)	2 (4)	3 (3)	3 (3)	1 (2)

a *P*=.04 when comparing groups based on years since graduation.

Overall, we can see in Table 3 that physicians intended to apply AI in medicine frequently (69/163, 42.3%) and believed that it would facilitate their work (140/153, 91.5%). Further, 49.1% (80/163) answered that AI would not interfere with the number of appointments, whereas one-third (54/163, 33.1%) of them believed that it would increase the number of appointments. They also believed that it would be useful for patients’ diagnosis and management (106/163, 65%).

Table 4 details the answers for questions 25-30. The answers revealed that physicians perceived that AI algorithms would not replace them but rather be 1 more source of information (105/163, 64.4%) and would not alter their financial gain (94/163, 57.7%). In the event of a diagnosis or conduct disagreement between physicians and the AI solution, we proposed 2 scenarios. In the first scenario, AI algorithms and physicians had the same accuracy rate for a defined task, and in the second scenario, AI had a better accuracy rate than physicians. We asked the physicians what should be done in each case. In the former case, physicians were divided between “asking for a third opinion” (86/162, 53.1%) or that “the medical opinion should be followed” (72/162, 44.4%). In the latter case, the majority (108/160, 67.5%) chose to request a third opinion. As for legal responsibility, most individuals (88/159, 55.3%) answered that it should be shared between the AI algorithm’s manufacturer and the physicians and hospitals. Further, 78.5% (128/163) responded that AI solutions should have the stamp of a regulatory governmental agency. No statistical differences in answers were found between groups based on years since graduation or sex, but we can see a trend for female physicians being more favorable toward governmental regulation (*P*=.055; not statistically significant).

**Table 4.** Effects of artificial intelligence (AI) solutions on the routine of medical work among those who answered the opinion questionnaire at the Hospital Israelita Albert Einstein (questions 25-30).

Questions and responses		Years since graduation, n (%)			Sex, n (%)	
		≤10 (n=12)	11-20 (n=46)	>20 (n=105)	Male (n=108)	Female (n=54)
**25. Physician’s replacement in medical specialties based on imaging? (radiology, dermatology, pathology, etc)**						
	Totally	1 (8)	2 (43)	2 (2)	5 (5)	0 (0)
	Partially	2 (17)	15 (33)	34 (32)	37 (34)	14 (26)
	One more source of information	9 (75)	28 (61)	68 (65)	64 (59)	40 (74)
	Not alter	0 (0)	1 (2)	0 (0)	1 (1)	0 (0)
	Do not know	0 (0)	0 (0)	1 (1)	1 (1)	0 (0)
**26. Financial gain**						
	Increases	5 (42)	5 (11)	12 (11)	15 (14)	7 (13)
	Decreases	1 (8)	7 (15)	18 (17)	19 (18)	7 (13)
	Not altered	6 (50)	27 (59)	61 (58)	61 (56)	33 (61)
	Do not know	0 (0)	7 (15)	14 (13)	13 (12)	7 (13)
**27. What to do if there is a disagreement between AI and physicians (supposing accuracies are equal)? (>20 years since graduation: n=104; female: n=53)**						
	Favors physicians’ opinion	5 (42)	23 (50)	44 (42)	52 (48)	20 (38)
	Favors AI’s opinion	0 (0)	0 (0)	1 (0)	1 (1)	0 (0)
	Request a third opinion	7 (58)	23 (50)	56 (54)	52 (48)	33 (61)
	Do not know	0 (0)	0 (0)	3 (3)	3 (3)	0 (0)
**28. What to do if there is a disagreement between AI and physicians (supposing AI accuracy is greater than physicians’)? (>20 years since graduation: n=102; male: n=107; female: n=52)**						
	Favors physicians’ opinion	4 (33)	9 (20)	16 (16)	22 (21)	7 (13)
	Favors AI’s opinion	0 (0)	8 (17)	10 (10)	12 (11)	6 (12)
	Request a third opinion	8 (67)	29 (63)	71 (70)	69 (64)	39 (75)
	Do not know	0 (0)	0 (0)	5 (5)	4 (4)	0 (0)
**29. Legal liability (>20 years since graduation: n=101; male: n=106; female: n=52)**						
	Physician only	3 (25)	10 (22)	32 (32)	35 (33)	9 (17)
	AI only	1 (8)	1 (2)	6 (6)	5 (5)	3 (6)
	Shared equally	7 (58)	32 (70)	49 (49)	56 (53)	32 (62)
	Do not know	1 (8)	3 (7)	14 (14)	10 (9)	8 (15)
**30. Governmental regulation** ^**a**^						
	Yes	10 (83)	33 (72)	85 (81)	81 (75)	46 (85)
	No	0 (0)	7 (15)	10 (10)	15 (14)	2 (4)
	Do not know	2 (17)	6 (13)	10 (10)	12 (11)	6 (11)

a *P*=.055 when comparing male and female physicians.

## Discussion

### Principal Findings

We conducted a web-based survey study among physicians in a large hospital in Brazil to seek their opinions about the use of AI solutions in medical practice. To our knowledge, this is the first survey to interrogate physicians’ expectations, fears, and opinions of AI use in medicine in Brazil. Our target population was not intended to represent the entire population of Brazilian physicians. Even so, a survey performed in a single, large private hospital can be a way of drawing attention to and starting a debate about this new subject in medicine among our physicians, as well as capturing their expectations on the topic, as AI solutions for health care workers are scarce, very limited in range, and not widespread among specialties in our reality.

Perhaps because this subject is not yet present in daily practice to most of the physicians in our study, there was a low rate of response. However, according to the HIAE marketing department, it was considered a typical respond rate since physicians usually do not respond to questionnaires in general. We wanted to extend the time frame by a couple of months for the study, but the department responsible for medical communication was concerned about overwhelming the physicians with too much electronic information. Thus, we followed the hospital’s regular modus operandi.

We chose to analyze 2 different aspects: first, the number of years since graduation, divide in 3 groups: ≤10, 11-20, and >20 years; and second, sex. This was based on our personal experience and the common knowledge that younger individuals are keener to use technology applications than older individuals in general. Thus, individuals with >20 years since medical graduation would be older and might have a different approach toward AI applied to medicine than younger individuals. Additionally, female and male physicians could have different perceptions about it.

Our responders were mostly from the private sector and male and belonged to different medical areas and age groups, which was important to investigate a broader perception of medical AI. Most of the studies in medical AI acceptance were focused on specific areas, such as radiology [5-8], dermatology [1,9-11], and ophthalmology [12-14], which could be more affected than others by the adoption of AI solutions. Thus, it is important to have more general views about the topic. Our group of responders was very heterogeneous but may reflect the frequency of different specialties in the mailing list of the hospital. Thus, it would be expected to have more answers from pediatricians than psychiatrists, as an example.

We found that physicians who graduated more recently (≤10 years) showed intention to adopt AI solutions more frequently than physicians who graduated >10 years ago, likely because younger individuals have lived much of their lives using technology solutions, trusting them and relying on them for many daily tasks, and have used them more than older individuals. Furthermore, modifying old habits can be more difficult than incorporating new ones in early stages. Newly graduated medical students could already have learned about the use AI solutions in medicine, whereas older physicians would not be inclined to modify their habits as readily. Another statistically significant difference found was the more favorable opinion from female physicians about the use of AI solutions by nonphysician hospital staff. Male physicians were more conservative in that aspect, with higher number of answers against or totally against it.

Our results demonstrate that, overall, physicians have positive expectations about the use of AI in clinical practice, but they also have some concerns. Their answers indicated that medical use of AI solutions hopefully will facilitate their work and be useful for diagnoses, management, and exam interpretation. We saw a tendency for a less favorable opinion on AI for actions that are thought to be the core of physicians’ responsibility. The opinion on the use of AI was 91.5% (150/164) favorable for aiding exams interpretation, 87.2% (143/164) for diagnosis, and 85.5% (140/164) for management. One-third (54/163, 33.1%) believed that the number of appointments performed by them, overall, would increase, probably by increasing the speed in making diagnosis or management decisions. Even so, they indicated that AI solutions would not interfere with their financial gain.

Probable benefits of AI solutions included greater speed, accuracy, and cost reduction for the health care system. This finding is in accordance with previous studies. A recent systematic review that included 45 studies with physicians or medical students on clinical AI showed that >60% responders had optimistic outlooks in 84% of the studies [3]. There is also an expectation that AI in medical practice will meet the higher expectations of medical treatment and physicians and will increase the efficiency of clinical care, as AI is perceived as the next big thing that will sustainably change medicine toward precision and personalized medicine [15].

Our participants believed that they would not be replaced by AI but that it would become 1 more source of information to support their work. Although the current discourse in medical literature has shifted from replacement to support of medical activities, as seen in the idea of augmented intelligence, where humans and AI work together in functions that each of them do best [16], the adoption of AI also opens the possibility of transferring decision-making to other health professionals or patients. This possibility divided their opinion, with roughly half (78/163, 47.9%) of them being against it. Many comments revealed the fear of misinterpreting the results if no medical supervision was performed. Question 18 explored the effect of AI when used by the physicians in the role of patients themselves and clearly showed that it can generate a great deal of anxiety if a troublesome diagnosis was provided by AI without specialist supervision. In 1 article focusing on patients’ opinion about the use of medical AI, the patients appeared to be receptive to the use of AI for medicine if implemented in a manner that preserves the integrity of the human physician-patient relationship [1]. A review article on the convergence of human and AI poses an important statement on that matter: “Over time, marked improvements in accuracy, productivity, and workflow will likely be actualized, but whether that will be used to improve the patient-doctor relationship or facilitate its erosion remains to be seen” [17]. In China, another study showed that the general population is more distrustful of AI in medicine, unlike the overall optimistic views posed for AI, and that the level of trust is dependent on what medical area is subject to scrutiny [19]. Those aspects are also a big concern for our physicians: worsening of the patient-physician relationship was listed right after the fear of over relying on medical AI and causing them to lose their medical skills over time. The comments showed that the benefit of human contact and the detection of emotions by the physicians cannot yet be replaced. A study with more than 1000 physicians showed that the fear of medical AI was inversely associated with advanced or intermediate AI-specific knowledge when compared with basic knowledge [6].

Possible disagreements between AI algorithms and physicians in daily practice were also explored by the questionnaire. In both questions 27 and 28, physicians believed that a third opinion should be requested (86/162, 53% and 108/160, 67.5%, respectively). Nevertheless, when the accuracy of the AI is greater the physician’s (as supposed in question 28), the number of respondents who answered that the final decision should be the physician’s dropped from 44.4% (72/162) to only 18.1% (29/160), revealing that the informed performance of AI solutions is crucial for physicians to make decisions.

As for legal aspects, most physicians (88/159, 55.3%) believed that the liability should be shared between them and the AI solution, reflecting the idea that the developing an AI solution involves a serious action, which requires careful engagement of all stakeholders. According to a recent article [20], all players in this field, such as physicians, developers, and health care administrators, should recognize that the implementation of an AI solution is not just a technical challenge but rather presents ethical, legal, and social challenges as well. Thus, it is important to gather all stakeholders to develop AI collaboratively from outset to implementation and evaluation [20]. It is also clear that physicians require and trust the role of government agencies to regulate this field. This is corroborated by another study [21] that discusses how regulation will become increasingly important as more algorithms start to be used in real life. Regulatory approval should not only mitigate possible harms but also define a proper balance between risks and benefits and promote effective validation standards in real settings and innovations [21]. In our study, this was more important to female physicians than male physicians.

In conclusion, our survey explored the physicians’ views on AI medical solutions in a new global geographical area, showing a general positive attitude toward AI solutions, as well as some concerns, including regulation by governmental agencies, who should be using them, and the fear of physicians relying too much on them.

### Limitations

It is important to note that our web-based survey was a cross-sectional study and was based on physicians’ response from a single institution that has a particular interest in innovation and AI in medicine. Since the study was performed via email, physicians who answered the questionnaire could already be more likely to use technology in general. Additionally, the theme of the study was stated in the email’s subject; thus, those who are interested in it would be more likely to open the email and answer the questionnaire. Therefore, the results have to be considered within a possible bias for a more positive attitude toward technology and AI in health care if compared to all physicians working in other Brazilian hospitals.

## Supplementary material

10.2196/50803 Multimedia Appendix 1 Questionnaire used to survey the opinion of Hospital Israelita Albert Einstein physicians on artificial intelligence.

## Abbreviations

AI: artificial intelligence
CROSS: Checklist for Reporting Survey Studies
HIAE: Hospital Israelita Albert Einstein
ICF: informed consent form

## References

[1] Nelson CA, Pérez-Chada LM, Creadore A (2020). Patient perspectives on the use of artificial intelligence for skin cancer screening: a qualitative study. JAMA Dermatol.

[2] Maassen O, Fritsch S, Palm J (2021). Future medical artificial intelligence application requirements and expectations of physicians in German University hospitals: web-based survey. J Med Internet Res.

[3] Guo Y, Hao Z, Zhao S, Gong J, Yang F (2020). Artificial intelligence in health care: bibliometric analysis. J Med Internet Res.

[4] Plana D, Shung DL, Grimshaw AA, Saraf A, Sung JJY, Kann BH (2022). Randomized clinical trials of machine learning interventions in health care: a systematic review. JAMA Netw Open.

[5] Alsharif W, Qurashi A, Toonsi F (2022). A qualitative study to explore opinions of Saudi Arabian radiologists concerning AI-based applications and their impact on the future of the radiology. BJR Open.

[6] Blease C, Kaptchuk TJ, Bernstein MH, Mandl KD, Halamka JD, DesRoches CM (2019). Artificial intelligence and the future of primary care: exploratory qualitative study of UK general practitioners' views. J Med Internet Res.

[7] Chen M, Zhang B, Cai Z (2022). Acceptance of clinical artificial intelligence among physicians and medical students: a systematic review with cross-sectional survey. Front Med (Lausanne).

[8] Sharma A, Minh Duc NT, Luu Lam Thang T (2021). A consensus-based Checklist for Reporting of Survey Studies (CROSS). J Gen Intern Med.

[9] Huisman M, Ranschaert E, Parker W (2021). An international survey on AI in radiology in 1041 radiologists and radiology residents part 2: expectations, hurdles to implementation, and education. Eur Radiol.

[10] Huisman M, Ranschaert E, Parker W (2021). An international survey on AI in radiology in 1041 radiologists and radiology residents part 1: fear of replacement, knowledge, and attitude. Eur Radiol.

[11] Ooi SKG, Makmur A, Soon AYQ (2021). Attitudes toward artificial intelligence in radiology with learner needs assessment within radiology residency programmes: a national multi-programme survey. Singapore Med J.

[12] Polesie S, Gillstedt M, Kittler H (2020). Attitudes towards artificial intelligence within dermatology: an international online survey. Br J Dermatol.

[13] Pangti R, Gupta S, Gupta P, Dixit A, Sati HC, Gupta S (2022). Acceptability of artificial intelligence among Indian dermatologists. Indian J Dermatol Venereol Leprol.

[14] Cho SI, Han B, Hur K, Mun JH (2021). Perceptions and attitudes of medical students regarding artificial intelligence in dermatology. J Eur Acad Dermatol Venereol.

[15] Scheetz J, Rothschild P, McGuinness M (2021). A survey of clinicians on the use of artificial intelligence in ophthalmology, dermatology, radiology and radiation oncology. Sci Rep.

[16] Zheng B, Wu MN, Zhu SJ (2021). Attitudes of medical workers in China toward artificial intelligence in ophthalmology: a comparative survey. BMC Health Serv Res.

[17] Valikodath NG, Al-Khaled T, Cole E (2021). Evaluation of pediatric ophthalmologists' perspectives of artificial intelligence in ophthalmology. J AAPOS.

[18] SurveyMonkey.

[19] Ye T, Xue J, He M (2019). Psychosocial factors affecting artificial intelligence adoption in health care in China: cross-sectional study. J Med Internet Res.

[20] Carter SM, Rogers W, Win KT, Frazer H, Richards B, Houssami N (2020). The ethical, legal and social implications of using artificial intelligence systems in breast cancer care. Breast.

[21] Allen TC (2019). Regulating artificial intelligence for a successful pathology future. Arch Pathol Lab Med.

