# Supplementary material for: Medical Expectations of Physicians on AI Solutions in Daily Practice: Cross-Sectional Survey Study
Source: JMIRx Med. 2024 Mar 25;5:e50803. doi: 10.2196/50803 (PMC11080601; doi:10.2196/50803)
Supplement: Multimedia Appendix 1 [file xmed-v5-e50803-s001.docx]

**Multimedia Appendix 1**

Questionnaire used to survey the opinion of HIAE physicians on AI:

1) Informed Consent Form:

Hello,

You are being invited to participate in an opinion survey on new technologies that are emerging in the Health area, such as Telemedicine.

This study is being conducted by researchers from Hospital Israelita Albert Einstein (CAAE: 30749620.6.0000.0071) and aims to find out if participants are aware of what these technologies are, what they are or would be used for and the expectations they have about them.

There will be no direct benefits for the participants, but the answers will contribute to a better understanding of the issue and the need to disseminate more information in this regard.

The answers are all collected anonymously, so that the participant can express themselves freely.

There is no financial reward or reimbursement of proven costs for participating in the research.

By clicking on the word "ACCEPTED", you will be directed to the link with the questions. If you do not want to participate, click on "I DO NOT ACCEPT".

ACCEPTED

I DO NOT ACCEPT

2) What's your sex?

Female

Male

I don't wish to inform

3) How old are you?

18-25 years

26-35 years

36-45 years

46-55 years

56-65 years

>65 years

4) What is your highest level of education?

Degree in Medicine only

Residency or Specialization Internship

Master's or MBA

PhD degree

Post-doc

Associated Professor

Other

5) Which of these areas do you fit in best?

Pediatrics

Internal Medicine

Surgery

Orthopedics

Gynecology and Obstetrics

Psychiatry

Ophthalmology

Otorhinolaryngology

Dermatology

Radiology

Pathology

Management

Research

Other

6) How many years since your graduation?

< 5 years

5 -10 years

11-20 years

>20 years

7) Do you work as physician:

Mainly in the public sector

Mainly in the private sector

Equally in both sectors (public and private)

8) Do you work in the state capital, on the coast or inland?

Capital

Coast or Inland

9) Which state or Federal District? Please use the acronym

10) How do you consider your level of knowledge in ARTIFICIAL INTELLIGENCE?

Low

Intermediate

High

None

11) If there is an option to use ARTIFICIAL INTELLIGENCE for some task in your day-to-day life, how often would you choose to use them?

Never

Rarely

Sometimes

Often

Always

I do not know

12) Are you aware of any ARTIFICIAL INTELLIGENCE algorithms that have been approved for medical use?

Yes

No

I am not sure

13) What would be your opinion about the use of Artificial Intelligence by physicians to support the diagnosis of diseases, such as COVID-19?

I am totally in favor

I am in favor

Not in favor nor against

I am against

I am totally against

Other (comment):

14) What would be your opinion on the use of Artificial Intelligence by physicians to aid in the therapeutic management of diseases, for example, the ideal time to introduce corticosteroids in cases of COVID-19?

I am totally in favor

I am in favor

Not in favor nor against

I am against

I am totally against

Other (comment):

15) What would be your opinion on the use of Artificial Intelligence by physicians to aid in the X Ray interpretation, such as the degree of pulmonary involvement in Severe Acute Respiratory Syndrome (SARS)?

I am totally in favor

I am in favor

Not in favor nor against

I am against

I am totally against

Other (comment):

16) What would be your opinion about the use of Artificial Intelligence in a hospital environment by non-physicians (nurses, physiotherapists) to aid in the diagnosis or therapeutic management of diseases?

I am totally in favor

I am in favor

Not in favor nor against

I am against

I am totally against

Other (comment):

17) What would be your opinion about the use of Artificial Intelligence directly by the patient in aiding the diagnosis or therapeutic management of diseases? (For example, by taking a picture of the skin lesion and getting a diagnostic result whether it would be skin cancer or not)

I am totally in favor

I am in favor

Not in favor nor against

I am against

I am totally against

Other (comment):

18) In the case above, assuming you are the patient and your lesion was diagnosed as melanoma by the artificial intelligence, this would leave you:

Extremely anxious to schedule a dermatologist appointment immediately

Anxious to scheduling a dermatologist appointment whenever possible

I would not be shaken, but I would make an appointment just in case

I would not be shaken or make any appointment to discard it

I am a dermatologist

Other (comment):

19) What benefits do you believe may be more relevant in the use of artificial intelligence in Medicine? (you can tick up to 3 options)

Faster diagnosis or management

Greater accuracy in diagnosis or management

Reduction of health costs through earlier and/or more accurate diagnoses

Reduction in the number of subsidiary exams

Reduction in patient’s anxiety

Greater patient’s access to healthcare

Greater patient’s participation in the management of their own health

Other (comment)

20) What problems do you believe may be more relevant in the use of artificial intelligence in Medicine? (you can tick up to 3 options)

Loss of confidentiality of patient data

Making the doctor/patient relationship more distant

Use of medical data against the patient by employers and insurers

Providing wrong diagnoses and management

Leading the doctor to rely too much on its results and to the loss of his medical skills

Rising cost of healthcare in general

Lack of transparency in the process by which artificial intelligence produces its results

Other (comment)

21) How often do you imagine you would adopt artificial intelligence in your medical routine, if it were available, if it proved to be reliable in real-life scientific research and if it took up to 2 minutes of your time?

Never

Rarely

Sometimes

Often

Most of the times

Always

I do not know

22) Assuming an available, reliable AI algorithm that took up to 2 minutes of your time, do you imagine that artificial intelligence would:

Make your work easier

Make your job more difficult

Not change your work

I do not know

23) Regarding the number of appointments in your day-to-day work, do you believe that artificial intelligence solutions would:

Increase the number

Decrease the number

Not alter the number

I do not know

24) Regarding the utility of artificial intelligence in your day-to-day work, do you believe that:

It will be useful in aiding the diagnosis

It will be useful in helping to conduct

It will be useful in aiding the diagnosis and management

It will not help or hinder

It will hinder

I do not know

25) Regarding medical work that is based on image (radiologist, pathologist, dermatologist, among others) do you believe that artificial intelligence will mainly:

Replace the work of the doctor who works with imaging completely

Replace, in some cases, the work of the physician who works with imaging

It will be one more source of information added to what the doctor already has

It will not change the work of the physician who works with imaging

I do not know

26) Regarding the financial gain in your day-to-day work, do you believe that artificial intelligence solutions would:

Increase your gain

Decrease your gain

Not alter your gain

I do not know

27) Assuming a scenario where physicians and artificial intelligence solutions have the same accuracy rates for one diagnoses or therapeutic management. In case of disagreement between them, what should be done?

The physician's opinion must prevail

The artificial intelligence’s opinion must prevail

A third opinion should be requested

I do not know

None of above

Comment:

28) Assuming a scenario where artificial intelligence solutions have the greater accuracy rates for one diagnosis or therapeutic management than physicians in general. In case of disagreement between them, what should be done?

The physician's opinion must prevail

The artificial intelligence’s opinion must prevail

A third opinion should be requested

I do not know

None of above

Comment:

29) Regarding legal liability when an artificial intelligence algorithm was used to aid the diagnosis and/or therapeutic management of a patient by the physician, do you believe that:

Responsibility should rest solely with the physician

Responsibility should rest solely with artificial intelligence

Responsibility should be shared between the doctor and artificial intelligence

I do not know

None of above

Comment:

30) Do you think that artificial intelligence algorithms should be evaluated by a competent government body before being released for use?

Yes

No

I do not know
